# Wilson’s Cases of Puerperal Fever

**Published:** 1842-10-15

**Authors:** M. W. Wilson

**Affiliations:** Resident Physician


### MEDICAL EXAMINER.

NEW SERIES.

No. 42.] PHILADELPHIA, OCTOBER 15, 1842. [Vol. I.

*A Report of Cases of Puerperal Fever, illustrative of the Malignant Epidemic that prevailed in the Lying-in Department of the Philadelphia Hospital, during the months of March and April of* 1842. By M. W. Wilson, M. D., Resident Physician.

Susan Carroll, æt. 23, a robust Irish girl, was delivered of her first child on the 22d of March, after a severe labour of about eighteen hours duration, attended with a rupture of the perineum. After the labour terminated she came under my care. In order to induce union of the rupture, as speedily as possible, a sponge was introduced into the vagina above the injury, to absorb the fluids, and her knees were confined together by a bandage. The lochia appeared early on the first, the milk was established on the second day from her labour. The bowels were opened with a laxative on the third day, and the patient appeared to be doing very well. She had been seized with occasional attacks of paroxysms of pain in her abdomen, attended with abdominal teuderness, which always yielded to warm cataplasms. The sponge was withdrawn from the vagina daily and reintroduced.

On the 26th,—the fourth day of her confinement,—she had a rigor, followed by a return of the pain and tenderness in her abdomen. The pulse was 90 per minute, and corded ; tongue dry and slightly coated. She was directed to have forty leeches applied to the abdomen ; the cataplasms were continued, and the following draught was ordered to be taken at night.

℞. Olei Ricini, ℥ss.

Tinc. Opii, gtt. xxx.

27th. Patient slept well last night ; her bowels were gently moved once this morning ; pain and tenderness much ameliorated ; pulse quick and moderately full ; tongue dry, and more thickly coated, and chapped. The cataplasms were continued.

Evening. The pains come on in paroxysms, lasting a few minutes, and then entirely leave her. The abdominal tenderness continues, but less severe. The cataplasms were continued, and four grains of camphor with one-sixth of a grain of the sulphate of morphia were prescribed.

28th. Patient slept well ; the pain still recurs in paroxysms, but they are much less severe. She complains of some constant pain in the left iliac region. Pulse small and corded, about ninety. She was ordered thirty leeches to be applied over the seat of the pain, and to continue the cataplasms.

Evening. The abdomen is swollen, tympanitic, and painful. The pain is greatly relieved by firm pressure on the abdomen. Skin hot and dry ; pulse about one hundred. She was directed to drink water as hot as she could bear it, which was followed by almost immediate relief of the pain, and a profuse diaphoresis. The cataplasms were continued, and the anodyne repeated.

29th. Expression anxious; respiration oppressed; abdomen swollen; pain and tenderness increased; bowels moved several times during the night; pulse 120, corded; skin hot; tongue coated, dry, and dark coloured. She was directed to have fifty leeches applied to the abdomen, and the cataplasms to be continued, and the anodyne repeated at night.

30th. Countenance more anxious; dyspnœa increased; respiration about forty per minute; pulse 140, small and tense. Abdomen more distended; bowels opened frequently. She was ordered to have forty leeches applied to the abdomen, and the cataplasms continued; and also to take three grains of blue pill every two hours. Dr. Gillingham visited the patient, and advised the treatment to be continued.

Evening. All the symptoms are aggravated; the abdomen is enormously distended and tender. A rectum tube was introduced up the rectum, and some feces, with considerable flatus escaped, until a contraction of the bowels destroyed the tube. She was then directed an assafœtida enema. The epidermis was raised with Granville's "antidynous lotion," and the surface dressed with mercurial ointment.

31st. Pulse very quick and frequent. The abdomen is larger than it was before her confinement. Her bowels were not opened since yesterday. Directed camphorated mercurial ointment to be applied over the whole abdomen, and covered with a warm cataplasm, and the following enema was administered.

℞. Olei Terebinth., ʒvj.

Tinct. Cardamomi, ℥i.

Misturæ Assafœt., ℥iss. M.

No action followed, and a rectum tube was again introduced, through which some fluid and considerable flatus escaped.

She commenced sinking rapidly at two o'clock—sordes collected on the teeth; there was extreme dyspnœa and occasionally low muttering delirium. She died at six o'clock in the evening.

*Necroscopy eighteen hours after Death.*

On cutting into the peritoneum, a considerable quantity, perhaps two or three pints, of dark coloured serum, mixed with flakes of lymph was observed. The peritoneum was finely injected throughout its extent, of a dark red colour. The omentum majus was finely injected and covered over with tenacious lymph. The intestines, both large and small, were very much distended with gas, except the lower portion of the colon, which was much contracted. The mucous membrane was natural.

The *uterus* was of the ordinary size after confinement. The parietes were softened. The internal surface was lined with a dark grayish substance, very tenacious, and about two lines and a half in thickness. In some places it appeared to extend into the substance of the uterus. At the lower part the colour was of a dark red, and appeared very much like clotted blood.

Dr. Gerhard considered it nearly in a gangrenons state.

*Case* 2.—Sarah Spangler, æt. 30, a healthy Irish woman, was delivered of her sixth child on the 11th of April. Her labour was not very severe, but it was attended with almost incessant vomiting. She was thirty-six hours in labour. She came under my charge on the following day, and appeared to be doing very well. The lochia was as usual, and the milk was established during the day. There was, perhaps, more tenderness than usual over the abdomen, and she was directed to keep a warm cataplasm of flaxseed constantly applied. On the afternoon of the 13th, or the third day of her confinement, she had a severe rigor, followed by severe pain in the abdomen. An hour after the chill, when I saw her, the pulse was 120 per minute, small and corded ; skin was hot and dry, and the tongue dry and coated. The lochia had ceased. She was directed to continue the cataplasm and to take five grains of calomel with ten of the ipecac. and opium powder.

14th. Countenance anxious ; pulse small and feeble, 120 ; she complains of pain in the fore part of the head. The abdominal pain and tenderness have diminished ; the lochia appeared again this morning. She was directed to continue the cataplasm, and to take two ounces of the infusion of cinchona three times a day.

Evening. The patient is very much improved. The pulse is a little excited, about 100 per minute, and more full. Tongue is coated along the edges, and red in the centre. The lochia continues, and the milk is increased. The treatment to be continued.

15th. The patient is much better. The pulse is but very little excited ; she has no pain or tenderness in the abdomen. The bowels were opened in the night.

Evening. She had another rigor this afternoon, followed by a return of the pain and tenderness in the abdomen ; hot and dry skin, and small quick pulse, (120 per minute.) The tongue is still coated. The hot cataplasm was continued, and she was directed to take a tablespoonful of the following prescription every two hours.

℞. Liquor. Ammoniæ Acetat., ℥ivss.

Spts. Æther. Nitrici, ℥iss. M.

16th. Pulse 180, small and quick. The patient is very restless. She slept none last night. The abdomen swollen and tympanitic, and the pain and tenderness increased. The expression is very anxious ; tongue still coated. She was directed to have thirty leeches applied to the abdomen, and to continue the cataplasm ; and also to take the following prescription :

℞. Hydrarg. Chlorid. Mit., gr. x.
Pulv. Ipecac. et Opii, gr. xv. M.

Evening. Pulse 135, feeble ; the pain and tenderness have increased ; the abdomen is less distended ; countenance sunken. She was directed brandy and egg freely. The cataplasm was continued.

17th. Delirium ; countenance expressive, haggard ; pulse quick and frequent ; pain and tenderness increased ; bowels opened during the night. The treatment to be continued.

Evening. The patient has had several convulsions during the afternoon, all the symptoms have increased in severity ; sordes is collecting on the teeth. The treatment to be continued, and prescribed

℞. Ammon. Carbon. ʒi.

Spts. Æth. Sulph. Comp., ℥ss.

Mucilag. G. Acac., ℥vss.

M. Dose, tablespoonful every hour.

Sinapisms were directed to the extremities.

18th. Patient is almost pulseless ; low muttering delirium ; and the extremities are cold. She died at 11 o'clock.

*Necroscopy, twenty-four hours after Death.*

The cavity of the peritoneum contained about two pints of serum, with flakes of lymph. The peritoneum was finely, but unequally injected throughout its extent. The omentum majus was finely injected and covered with lymph. The uterus was of the usual size after labour. The parietes were attenuated and softened. The internal surface was lined with a dark tenacious mass, which in some places appeared to penetrate into the substance of the uterus. The large veins of the uterus were natural, the other viscera were healthy, except the stomach, which presented some bright red injection in the mucous membrane, near the cardiac orifice.

*Case* 3.—Henrietta Hoffman, æt. 25, was confined with her second child on the 8th of April. There was some adhesion of the placenta, with hourglass contraction of the uterus, which caused some difficulty in its delivery. It was finally accomplished by introducing the hand and separating the placenta from the uterus. She now came under my care. About three days previous to the labour she had an attack of erysipelas, which involved one side of the face, completely closing one eye, and implicating a part of the scalp. Her treatment had been saline laxatives, with local applications of flaxseed mucilage.

9th. Pulse about ninety per minute; the redness of the face is diminishing. There is considerable tenderness over the abdomen, and she complains of pain in the hypogastrium, which comes on in paroxysms. Her bowels were opened several times during the night. She was directed a warm cataplasm to the abdomen; and to take fifteen grains of the ipecac. and opium powder.

10th. Pulse eighty, soft, and moderately full. Abdomen less painful and tender. The redness of face is improving. The cataplasm to be continued and also the mucilage to the face.

Evening. Patient had rigors this afternoon, about an hour previous to my visit. Pulse 130; small and feeble; skin hot and dry; tongue coated, dry, and chapped; the redness of the face is now almost confined to the eyelid. She has some tenderness over the abdomen; the pain, which is severe, comes on in paroxysms. The lochia continue, the milk has ceased. The bowels were opened four times in twenty-four hours. She was directed to continue the treatment, and also to take the following prescription:

℞. Hydrarg. Chlor. Mit., gr. x,
Pulv. Ipecac. et Opii., gr. xv. M.

11th. Countenance anxious; pulse 100, feeble; tongue moist and creamy. The pain of abdomen continues to recur at intervals; abdominal tenderness has diminished. She was directed to continue the cataplasms, and to take two ounces of infusion of cinchona every four hours, and four ounces of wine daily, by advice of Dr. Gillingham.

12th. Pulse more full and soft, and less frequent, about 90; the pain and tenderness have greatly abated. The patient says she feels much better. The treatment was continued.

13th. Pulse nearly natural, except more feeble; tongue moist; no pain or tenderness; the bowels are regular. The redness of face is diminishing. The treatment was continued.

14th. Patient is improving. The treatment continued.

15th. Still improving.

Evening. She had another rigor this evening, followed by a return of the abdominal pain and tenderness, which was attributed to the patient leaving her bed and walking out of the ward in an undress, contrary to directions. The treatment was still continued.

16th. Countenance anxious; pulse 100, small and corded; tongue moist; abdominal pain and tenderness continue. The treatment was continued.

Evening. Pulse 120; the symptoms more severe. Directed to continue the treatment, and to take fifteen grains of the ipecac. and opium powder.

17th. Pulse 120; feeble; countenance very anxious. The pain has increased, and she refers it more to the right iliac region, than elsewhere. She is obliged to lie with her thighs flexed, to relax the abdominal muscles. She slept but little during the night. When the paroxysm of pain comes on, she screams out so as to alarm the patients in the adjoining ward. She was directed to continue the cataplasm, and to take the following prescription:

℞. Hydrarg. Chlorid. Mit., gr. xxxij.
Pulv. Ipecac. et Opii, ʒi.
M. Div. in Chart. No. iv.
Take one every four hours.

Evening. The patient is much easier. The treatment to be continued.

18th. She complains of very little pain, and no tenderness in the abdomen. The treatment was discontinued.

I now left the ward, but the patient continued to improve. She was considered as convalescent, and after this date required but little treatment. She has since left the house, quite well.

*Case* 4.—Sarah Smith, æt. 23, of full, plethoric habit; subject to convulsions of an epileptiform character; during gestation was confined of a stillborn child, April 12th, after a protracted labour of eight days duration. During her labour, between the paroxysms of pain, she appeared unusually cheerful, and occasionally had strange hallucinations. For twelve hours previous to the termination of labour, she fancied her child was born and secreted by the nurse, and she was only convinced of her error by a few severe pains, which terminated her labour. Immediately after the placenta came away, she was taken with rigors which lasted for upwards of an hour and a half. Hot drinks and hot applications were directed to the abdomen, and sinapisms to the extremities. Profuse perspiration followed the rigor; the pulse was full and strong, about 90; the hallucinations still continued. She was ordered fifty leeches to the temples, which gave some relief. In the afternoon, the abdomen was very painful, tender, and swollen. She was bled to eighteen ounces by the advice of Dr. Gillingham.

℞. Pulv. Ipecac. et Opii, ʒj. was prescribed at night, with orders to repeat it at 12 o'clock, if sleep should not be produced.

13th. Slept after the second powder was administered; the pulse is small, frequent, and moderately tense; tongue dry, and covered with a dark yellowish coat in the centre. The bowels were opened last night; the pain and tenderness continue in the abdomen; delirium persists; she begs of me to take the afterbirth away. The lochia have ceased. She was ordered ten leeches to the os uteri, and forty to the abdomen, and the following prescription:

℞. Hyd. Chl. Mit. gr. x.; P. Ipecac. et Opii, ℈j. M.

Hot cataplasms were continued to the abdomen.

Evening. Pulse about 100; very weak; tongue coated, dry and chapped, red along the edges; respiration hurried; abdomen greatly distended; complains of pain and tenderness over the abdomen; the delirium continues the same. The cataplasm to be continued, and the following prescription directed:

℞. Ammon. Carb. ʒj.

Spts. Æth. Sulph. Comp. ℥ss.

Aquæ Menth. ℥vss.

M. Dose, a half ounce every hour.

She was ordered brandy and egg freely; sinapisms to extremities; the delirium increased, and she would take no medicine. Stimulating injections were ordered, and sinapisms over the abdomen. She continued to sink until 5 o'clock in the morning, when she died. Her friends prevented an examination of the body.

*Case* 5.—Fanny Tillsman, a colored woman, æt. 30, of stout, robust frame, was delivered of her sixth child on the evening of the 8th of April, 1842. She was left on the lying-in bed while her physician was absent for a few minutes, with instructions not to leave it, but neglecting these instructions she went to the close stool, and while straining on it, a sudden contraction of the uterus expelled the child and ruptured the perineum. On the 9th, when she came under my charge, her expression was anxious; pulse small and frequent, about 100; tongue red in the centre and coated along the edges; great abdominal tenderness, particularly in the hypogastric region, and perhaps a little swelling. Her bowels were open frequently during the night. She had been troubled with a looseness of the bowels for about twenty-four hours previous to labour, with some tenesmus. She was ordered a hot cataplasm to abdomen, and Pulvis Ipecacuanhæ et Opii, gr xv. every three hours.

Evening. Pulse small, frequent, and moderately tense; the abdomen is very much enlarged; pain and tenderness are increased; the bowels were opened three times since morning. She was ordered forty leeches to the abdomen; to continue cataplasm and a pill composed of

℞. Hydrarg. Chlor. Mit. gr. j.
Pulv. Ipecac. et Opii, gr. iij.
Every three hours.

10th. Pulse 120, feeble; tongue coated as yesterday, more glossy in the centre; bowels opened very frequently; pain and tenderness in abdomen increased. The treatment to be continued.

Evening. All the symptoms aggravated; voice very indistinct; the lochia has stopped; the milk has diminished in quantity. She was ordered six ounces of wine a day in gruel, and the following mixture. The cataplasm to be continued.

℞. Ammon. Carb. ʒi.

Spts. Æth. Comp. ℥ss.

Lac Assafœt. q. s. ℥vi.

M. Fiat mist. Dose, a table spoonful every two hours.

11th. Pulse 140, very feeble; abdomen greatly distended, very painful and tender on pressure; bowels open very frequently, with bloody stools. Treatment continued, with enema opii every four hours.

Evening. Pulse more full and strong, about 120 per minute; the swelling of abdomen is subsiding; no change in the character or frequency of the stools. The treatment to be continued.

12th. Pulse very frequent and feeble; tongue still coated; sordes collecting on the teeth; drops of cold perspiration are standing on her face; abdomen less painful, and less tympanitic; extremities cold; bowels opened but once last night. She was directed sinapisms to the extremities, and brandy and egg freely, and, also, an enema of six drachms of Oleum Terebinthininæ in mucilage, and to be repeated in two hours.

Evening. All the symptoms aggravated, but tympanitis much less; pulse extremely weak and frequent; expression haggard; intellect clear. She died in the night.

*Necroscopy twenty-eight hours after Death.*

External appearance. Abdomen very little, if at all distended; breasts small, and milk exudes when cut into; no emaciation. On opening the peritoneum, about a pint and a half of reddish-brown serum escaped. The peritoneum exhibits a patch of dark-red colour, and finely injected in about four inches square at the region of the umbilicus. The peritoneal surface of the ileum about four feet from the ileo-cœcal valve is of a dark-brown colour, extending downwards two feet, corresponding in position with the fundus of the uterus, and lying immediately against it. Elsewhere the intestines are healthy on the peritoneal surface.

The stomach contained some fluid of a dark muddy appearance. Some patches of bright red injection were observed in the mucous membrane, and they extend down the duodenum. The mucous membrane is not softened. The ileum, about eight feet above the ileo-cœcal valve, shows traces of inflammation, which increase for four feet in intensity. At this place the mucous membrane is thickened, somewhat softened, and there is a deposition of blood beneath. This appearance, internally, corresponds with a dark-brown colour, observed on the peritoneal coat of the intestines, and ends as it commenced, very abruptly. Below this, the intestine is very much attenuated to the end of the ileum. The glands of Peyer are ulcerated, resembling the state of these glands in typhoid fever. The mucous membrane is very much thickened, and covered with lymph throughout its whole extent. It resembles a tissue studded with warts.

The liver is soft and pale. The spleen, kidneys and pancreas are healthy. The uterus is of the usual size after labour; parietes, perhaps a little softened, internally it is lined with a dark red substance apparently extending into the substance of the uterus; it varies in thickness from two and a half to five lines. In color and consistence it bears resemblance to coagulated blood on the surface, but becomes more firm below, as we remove it. The ovaries are perfectly healthy.

Several children died in the lying-in ward, of peritonitis during the prevalence of this epidemic. One of them died five days after birth, without any symptoms of disease, until a few minutes before death. It took the breast fifteen minutes previous to death, and appeared perfectly well. On examination of the body, the peritoneum was found of a dark red colour, and finely injected throughout its whole extent, and the liver was much congested with blood; all the other viscera were healthy. The mother of this child escaped the epidemic. Two other children died, after a few days illness, one five, and the other seven days after birth. On examination the appearances presented were the same as in the first. The mothers of both these children had the fever; one of them died. There were two other cases that died after I left the wards, very much like those detailed above.

From an examination of these cases, it is obvious that large depletion could not be practised with benefit. The low form of the disease which was apparent from the feeble and frequent pulse, and also the known typhoid tendency of the house, as indicated by the presence of erysipelas and typhus fever, went far to contra-indicate its use.

The state of the patient in whom bleeding was practised, was considered much more favourable for its employment than that of any case reported above. But the patient died, and the duration of the disease was shorter than of any other patient treated. It was also practised in another case, not included in the above report, immediately after reaction from the chill, and before the pulse exceeded 90 in frequency. She was a fine robust young Irish woman, and her pulse decidedly full and strong. The case, however, like the others, terminated fatally, and in a shorter time than the majority of the cases included in this report. I am unable to give the complete history of this case, as she passed from my care before death, by a change in the wards.

